# Varicella vaccine without human serum albumin versus licensed varicella vaccine in children during the second year of life: a randomized, double-blind, non-inferiority trial

**DOI:** 10.1186/s12887-016-0546-5

**Published:** 2016-01-13

**Authors:** Roman Prymula, Robert Simko, Michael Povey, Andrea Kulcsar

**Affiliations:** Faculty of Military Health Sciences, University of Defence, Trebesska 1575, 50001 Hradec Kralove, Czech Republic; Primary Care Paediatric Praxis, No 8 Miskolc, Hungary; GSK Vaccines, Avenue Fleming 20 B-1300, Wavre, Belgium; Szent László Hospital, Gyali Street 5-7, Budapest, 1097 Hungary

**Keywords:** Varicella vaccine, Non-inferiority, Human serum albumin, HSA

## Abstract

**Background:**

GSK’s varicella vaccine contains human serum albumin (HSA) which is used to stabilize the virus and prevent immunogens from adhering to the injection vial walls. However, because HSA is derived from human blood, there is a theoretical risk that it might contain infectious agents which could be unsafe for humans. Given this concern, a study was undertaken to compare the immunogenicity and safety of a new formulation without HSA with the currently licensed varicella vaccine in the Czech Republic and Hungary.

**Methods:**

Healthy children aged 11–21 months received two doses of the varicella vaccine either with or without HSA. Antibody titres against varicella-zoster virus (anti-VZV) were measured 42 days after each dose, using an immunofluorescence assay (IFA, cut-off = 4dilution^−1^) and enzyme linked immunosorbent assay (ELISA, cut-off = 25 mIU/ml). Solicited local symptoms were recorded during a 4-day post-vaccination follow-up period; solicited general and unsolicited symptoms were recorded during a 43-day post-vaccination follow-up period and serious adverse event (SAEs) were recorded throughout the study.

**Results:**

Of 244 children (mean age = 15.2 months [SD = 3.2]) vaccinated in the study, 233 (vaccine without HSA *N* = 117; vaccine containing HSA *N* = 116) formed the according-to-protocol immunogenicity cohort. Observed seroconversion/seroresponse rates were >98 and 100 %, 42 days after doses 1 and 2, respectively. The rates were within the same range in both groups, irrespective of the testing assay. The varicella vaccine without HSA was non-inferior to the licensed vaccine in terms of anti-VZV antibody Geometric Mean Titre/Concentration ratio (1.12 [95 % CI:0.86–1.46] by IFA; 1.12 [95 % CI:0.93–1.33] by ELISA) approximately six weeks after the first dose of the 2-dose vaccination course. The incidence of solicited and unsolicited symptoms was similar after both vaccines; low-grade fever was numerically higher after the first dose of the varicella vaccine without HSA. Seven SAEs were reported, none of which were fatal or considered to be vaccine-related.

**Conclusions:**

The first dose of a new varicella vaccine without HSA was immunologically non-inferior to the licensed varicella vaccine. After two doses, both vaccines had acceptable safety profiles in children aged 11–21 months in the Czech Republic and Hungary.

**Trial registration:**

NCT00568334, registered on 5 December 2007 (www.clinicaltrials.gov).

## Background

In children, primary infection with varicella-zoster virus (VZV) causes chicken pox [1], a highly contagious but mostly benign disease. It can, however, be associated with a range of serious complications such as secondary bacterial infections, pneumonia, cerebellar ataxia, encephalitis and Reye’s syndrome [1]. In adolescents and adults, primary VZV infection causes greater morbidity and mortality [2]. Following primary VZV infection, the latent virus persists in the central nervous system and can be reactivated in later life to cause herpes zoster (HZ) or shingles [2, 3]. Complications of HZ include herpes ophthalmicus, cutaneous dissemination, pneumonia and other central nervous system, pulmonary, hepatic and pancreatic complications [4].

In Europe, over 90 % of children contract chicken pox in the first 12 years of life [5–9]. Although the reported incidence of VZV-related deaths (≤0.05/100,000 population/year) and hospitalizations (1.1–18.7/100,000 population/year) in European countries is low, the burden of disease is nevertheless significant [10–12].

Two monovalent varicella vaccines are currently available in Europe: *Varilrix*™ (GSK Vaccines) and *Varivax*™ (Merck & Co. Inc), both of which are derived from the Japanese Oka strain. Placebo-controlled clinical trials have demonstrated that both vaccines are immunogenic, safe and efficacious [13, 14]. A recent observer-blind study in Europe showed 65.4 % efficacy for one varicella vaccine dose at preventing confirmed varicella of any severity, and 90.7 % efficacy at preventing moderate to severe cases during 3 years of follow-up [15].

Since the World Health Organization recommendation for routine vaccination against childhood VZV infection in 1998 [16], several countries have implemented either of the varicella vaccines into their national immunization programs [17–24]. By 2008, the human serum albumin (HSA)-containing varicella vaccine (*Varilrix™*) was licensed in 92 countries [25].

HSA is incorporated in live, attenuated vaccines to stabilize the virus and prevent immunogens from adhering to the walls of the injection vial. However, because HSA is a protein derived from human blood, there is a theoretical risk that it might contain infectious agents [26] which could make it unsafe for humans. Given this concern, the manufacturing process has been modified to remove HSA from the vaccine stabilizer added towards the end of production. This is in line with the European Medicines Agency recommendation to eliminate all blood-derived products of human origin from vaccine manufacturing [27]. This study was therefore undertaken to confirm that the varicella vaccine without HSA was immunologically non-inferior to the licensed varicella vaccine. The safety profile of both vaccines was also assessed despite the process change.

## Methods

### Study design and subjects

This phase II, non-inferiority study was conducted at 14 centres (offices of primary care physicians and general practitioners) in the Czech Republic (2 centres) and Hungary (12 centres) between November 2007 and April 2008 (NCT00568334). The primary care physicians recruited children and depending on the centre, either the physician/nurse administered the vaccine. The study was carried out in a double-blind manner, meaning that the investigators, study staff and parents/guardians of children were unaware of the vaccine given.

Healthy children 11–21 months of age were enrolled and randomized (1:1) to receive either two doses of varicella vaccine without HSA (Group A) or two doses of varicella vaccine containing HSA, *Varilrix*™ (Group B) which is licensed for the prevention of varicella in healthy children from 9 months of age. The selection criteria in this study were similar to previous studies conducted with the licensed vaccine [28, 29]. The two doses were administered 42–56 days apart. Children were excluded from the study if they had received any live attenuated viral routine vaccines or investigational drug/vaccine within 30 days or immunosuppressant/immune modifying drugs/blood-derived products within six months of receiving the study vaccine. They were also excluded if they had been previously exposed to VZV or had: any confirmed or suspected immunosuppressive condition; a family history of immunodeficiency; chronic illnesses, allergies to any vaccine components or an acute disease at the time of enrolment. In order to account for the multiple routine vaccinations recommended at this age, children were allowed to receive routine inactivated vaccines up to eight days before each study vaccination so as to not interfere with the reactogenicity assessment after varicella vaccination. It was advised that children participating in the study should avoid the use of aspirin for six weeks after vaccination as Reye’s syndrome has been reported following the use of aspirin during natural varicella infection.

All study-related documents including the study protocol were reviewed and approved by the Pardubice Regional Hospital and the Regional Hospital Nachod for the two participating centres, respectively, in the Czech Republic and by the Egészségügyi Tudományos Tanács Klinikai Farmakológiai Etikai Bizottság for the 14 participating centres in Hungary. The study was conducted in accordance with Good Clinical Practice, the Declaration of Helsinki and all applicable regulatory requirements. Parents/guardians provided written informed consent before any study procedures were performed.

### Study vaccines

Both varicella vaccines were developed and manufactured by GSK Vaccines, Belgium. The potency of the Oka/RIT strain in the licensed varicella vaccine was 10^4.4^ plaque forming units (pfu)/0.5 mL compared with 10^4.3^ pfu/0.5 mL in the formulation without HSA. Both vaccines were presented as lyophilized pellets, which were reconstituted with the supplied diluent before subcutaneous injection in the left upper arm.

### Immunogenicity

Blood samples were collected immediately before vaccination and 42 days after each vaccine dose. Antibody titres against VZV (anti-VZV) were measured using two assays: a commercial enzyme linked immunosorbent assay (ELISA kit, *Enzygnost™*, Dade Behring, Marburg, Germany; assay cut-off = 25 mIU/ml) and an immunofluorescence assay (IFA, *Virgo™*, Hemagen Diagnostics, Columbia, MD, USA; assay cut-off = 4dilution^−1^).

### Safety

Parents/guardians used diary cards to record solicited local and general symptoms and unsolicited adverse events. Solicited local symptoms around the injection site (pain, redness and swelling) were recorded for four days after each dose and solicited general symptoms (fever [axillary temperature ≥37.5 °C/rectal temperature ≥38 °C] and rash/exanthema) were recorded for 43 days after each dose. Body temperature was measured daily via the rectal/axillary route for eight days after each vaccine dose. Between days 8–42, the presence of fever was monitored using a temperature-sensitive forehead thermometer (*FeverScan*™, LCR Hallcrest, UK). If the forehead thermometer indicated fever, temperature was accurately measured using a thermometer and recorded.

The intensity of solicited symptoms was graded on a scale of 0–3. Grade 3 solicited symptoms were defined as: child cried when limb was moved or a spontaneously painful limb (pain); injection site surface diameter >20 mm (redness and swelling); axillary temperature >39 °C/rectal temperature >39.5 °C (fever) and ≥150 lesions (rash/exanthema).

Unsolicited symptoms were recorded for 43 days after each dose and were classified as Grade 3 if they prevented everyday activity. Serious adverse events (SAEs) were recorded throughout the study.

### Statistical analysis

All statistical analyses were performed using SAS 9.1 and Proc StatXact 7.0 [30, 31].

The primary objective of the study was to demonstrate non-inferiority of the first dose of the varicella vaccine without HSA when compared to the licensed vaccine. This was achieved if the lower limits (LL) of the two-sided standardized asymptotic 95 % confidence intervals (CI) for the Geometric Mean Titre (GMT) ratio between the vaccine without HSA and the licensed vaccine was ≥0.5 (measured by IFA) and the Geometric Mean Concentration (GMC) ratio between two groups was ≥0.67 (measured by ELISA). The sample size calculated based on non-inferiority of immunogenicity and a sample size of approximately 100 subjects for each treatment group rendered 92 % power to the analyses. The analysis of safety data was descriptive.

A central randomization system, using a minimization algorithm, provided each child with a unique treatment number. After randomization, a central blocking scheme was used to ensure that the balance between treaments was maintained.

Immunogenicity analysis was conducted on the according-to-protocol (ATP) cohort for immunogenicity, which included all subjects for whom both pre- and post-vaccination data were available for both vaccine doses, who were seronegative (anti-VZV antibody titre below the assay cut-off) for anti-VZV antibodies before vaccination, and complied with study procedures. The analysis of safety was conducted on the total vaccinated cohort (TVC) which included all subjects who received at least one dose of study vaccine.

Seroconversion (IFA data) was defined as an anti-VZV antibody titre ≥ the assay cut-off value (4dilution^−1^) in previously seronegative subjects. Seroresponse (ELISA data) was defined as an antibody concentration ≥ the assay cut-off (25 mIU/mL) in previously seronegative subjects.

The percentage of subjects who showed seroconversion or seroresponse after both vaccine doses was calculated with 95 % CI. The corresponding anti-VZV GMT/GMC values were calculated with 95 % CI by taking the anti-log of the mean of the log of titre/concentration transformations. Values below the cut-off were given arbitrary values of half the cut-off for the purpose of this study. The GMT/GMC ratio was calculated using an analysis of variance. The ratio and 95 % CI were calculated as an exponential transformation of the values. A two-sided standardized asymptotic 95 % CI was calculated on the difference in the percentage of subjects with anti-VZV antibody concentrations ≥50 mIU/ml.

## Results

### Baseline characteristics

**Fig. 1:**
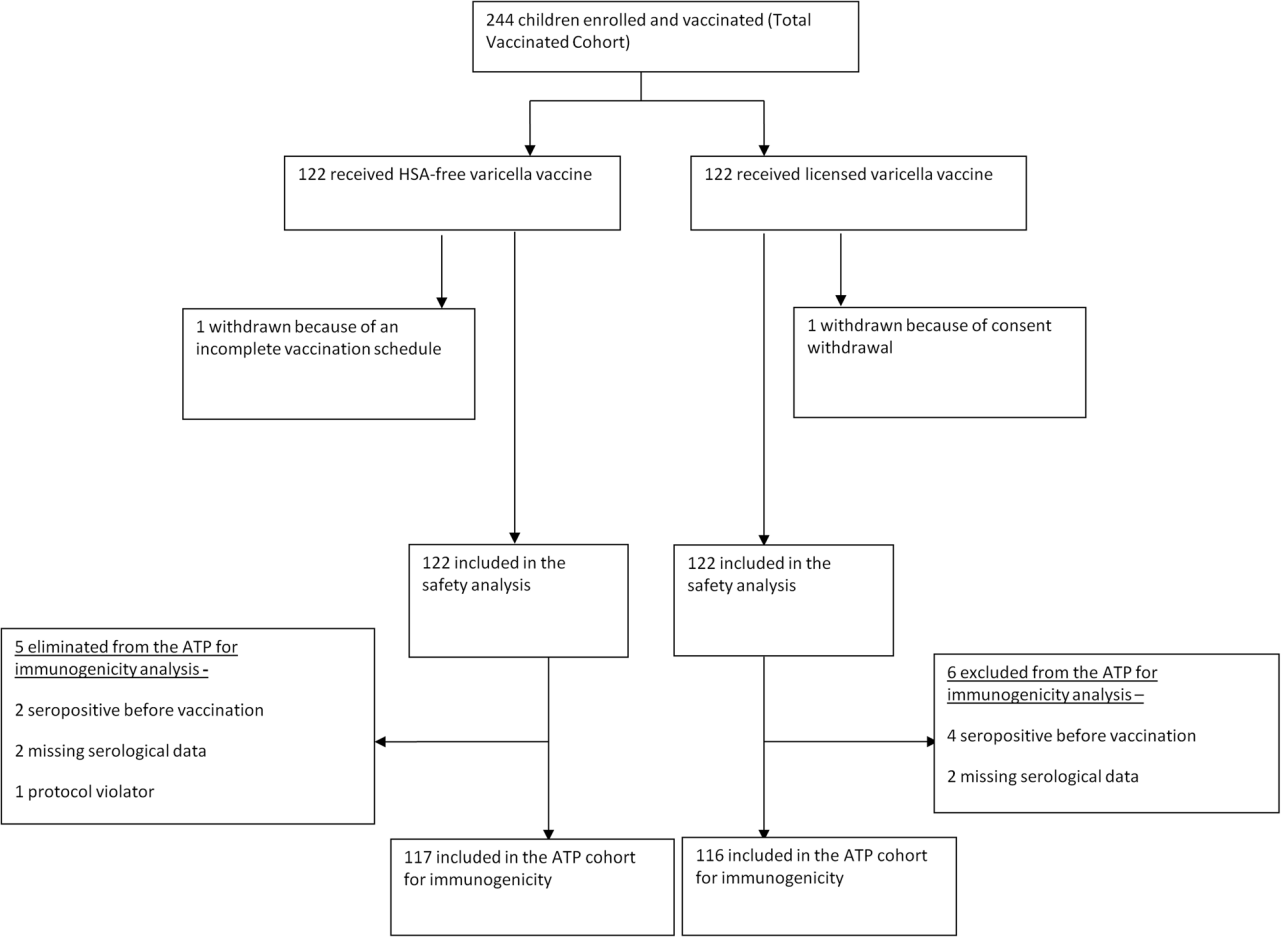
Participant Flowchart

A total of 244 subjects were enrolled (122 in each group) of whom 233 were included in the ATP immunogenicity cohort (Group A = 117; Group B = 116; Fig. 1). The baseline characteristics were similar between the groups (Table 1). The mean age of the children in the TVC was 15.2 months (standard deviation [SD]: ±3.2), 51.2 % were male, and all were Caucasian.

**Table 1:** Baseline characteristics (total vaccinated cohort *N* = 244)

Characteristic		Group A (*N* = 122)	Group B (*N* = 122)	Total (*N* = 244)
Age (months)	Mean	15.6	14.8	15.2
	SD	3.3	3.1	3.2
Gender (n [%])	Male	63 (51.6)	62 (50.8)	125 (51.2)
	Female	59 (48.4)	60 (49.2)	119 (48.8)

Group A: varicella vaccine without HSA administered according to a two-dose regimen

Group B: licensed varicella vaccine administered according to a two-dose regimen

N: total number of subjects

n (%): number (percentage) of subjects in each category

SD: standard deviation

### Immunogenicity

The proportion of initially seropositive individuals was 0.8 % in Group A and 3.3 % in Group B when antibody titres/concentrations were analyzed by IFA, compared with 0.8 % in both groups when analysed using ELISA.

Six weeks post-dose-1, the seroconversion rates, as measured by IFA, were similar in Group A (98.3 % [95 % CI: 93.9–99.8 %]) and B (99.1 % [95 % CI: 95.3–100 %]) (Table 2). Post-dose-2 seroconversion rates were 100 % in both groups. After dose-1, 90.5 % (95 % CI: 83.7–95.2 %) subjects in Group A and 89.6 % (95 % CI: 82.5–94.5 %) in Group B had concentrations of anti-VZV antibodies ≥50 mIU/ml.

**Table 2:** Immunogenicity of vaccine without HSA and licensed varicella vaccines six weeks after doses 1 and 2 (according-to-protocol immunogenicity cohort *N* = 233)

Dose	Group A Varicella vaccine without HSA			Group B Licensed varicella vaccine			Ratio (Group A/Group B) Post-dose 1
	*N*	SC % (95 % CI)	GMT (95 % CI)	*N*	SC (95 % CI)	GMT (95 % CI)	GMT (95 % CI)
IFA							
6 weeks Post-dose 1	116	98.3 (93.9; 99.8)	172.6 (141.6; 210.3)	115	99.1 (95.3; 100)	154.3(128.7; 185.0)	1.12 (0.86; 1.46)
6 weeks Post-dose 2	115	100 (96.8; 100)	1452.5 (1240.7; 1700.5)	112	100 (96.8; 100)	1395.4 (1183.0; 1645.9)	
Dose	*N*	SR (95 % CI)	GMC (95 % CI)	*N*	SR (95 % CI)	GMC (95 % CI)	GMC (95 % CI)
ELISA							
6 weeks Post-dose 1	116	98.3 (93.9; 99.8)	123.5(107.9; 141.4)	115	98.3 (93.9; 99.8)	110.7 (98.4; 124.6)	1.12 (0.93; 1.33)
6 weeks Post-dose 2	116	100 (96.9; 100)	1013.6 (880.9; 1166.4)	114	100 (96.8;100)	999.2 (877.3; 1138.1)	

N: total number of subjects

95 % CI: 95 % confidence interval

SC %: seroconversion rate

SR %: Seroresponse rate

GMT: geometric mean antibody titre calculated on all subjects by IFA

GMC: geometric mean antibody concentration calculated on all subjects by ELISA

Criteria for non-inferiority:

The lower limit of the 95 % confidence interval (CI) for the GMT ratio (derived by IFA) between Group A and Group B is equal to or above the pre-defined clinical limit of 0.5

The lower limit of the 95 % CI for the GMC ratio (derived from ELISA) between Group A and Group B is equal to or above the pre-defined clinical limit of 0.67

Six weeks post-dose-1, the seroresponse rates as measured by ELISA, were 98.3 % in Groups A and B (Table 2). Six weeks post-dose-2, the seroresponse rates were 100 % in both groups.

The primary non-inferiority criteria post-dose-1 was achieved since the LL of the 95 % CI between the vaccine without HSA and licensed varicella vaccine were: 0.86 for the GMT ratio by IFA (i.e. ≥ the pre-defined clinical limit of 0.5); and 0.93 for the GMC ratio by ELISA (i.e. ≥ the pre-defined clinical limit of 0.67).

### Safety and reactogenicity

The incidence of solicited and unsolicited symptoms, 43 days post-dose-1 was: 76 % in Group A and 69.7 % in Group B; post-dose-2, the incidence was 71.9 and 68.6 %, respectively.

**Fig. 2:**
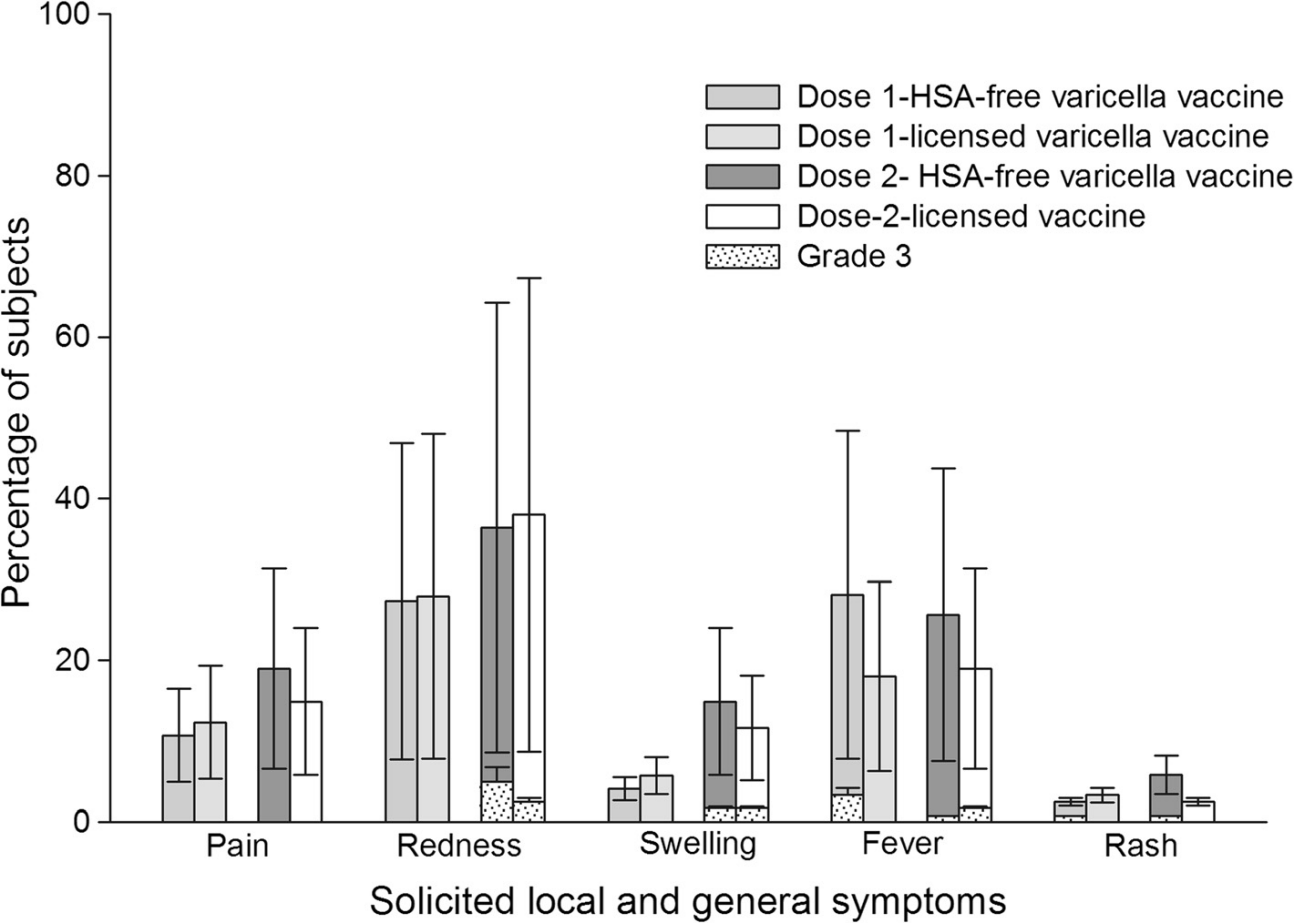
Solicited local (4-days post-vaccination) and general symptoms (43 days post-vaccination) (Total Vaccinated Cohort *N* = 244). %: percentage of subjects reporting the symptom at least once. 95 % CI: Exact 95 % confidence interval; Grade 3 pain: Cried when limb was moved/spontaneously painful; Grade 3 Redness/swelling >20.0 mm; Grade 3 Fever >39.0 °C; Grade 3 Rash >150 lesions

Injection site redness was the most common solicited local symptom (any and Grade 3) in both groups after both doses (Fig. 2). During the eight days of active follow-up after dose-1 when temperature was monitored daily, the fever rate in Group A was 28.1 % (95 % CI: 20.3–37.0 %) and 18.0 % (95 % CI: 11.7–26.0 %) in Group B. Post-dose-1, Grade 3 fever occurred in four subjects in Group A and two subjects in Group B. Post-dose-2, fever was recorded in 25.6 % (95 % CI: 18.1–34.4 %) of Group A subjects and 19 % (95 % CI: 12.4–27.1 %) of Group B subjects. Grade 3 fever, post-dose-2 occurred in one subject in Group A and two subjects in Group B. The observed incidence of fever during the 43 days of follow up post-dose-1 was also numerically higher in Group A (52.9 % [95 % CI: 43.6–62 %]) as compared with Group B (42.6 % [95 % CI: 33.7–51.9 %]) (Fig. 2), although the exact 95 % CIs rates were overlapping.

During the 43-day follow-up period post-dose-1, rash/exanthema was seen in 2.5 % (95 % CI: 0.5–7.1 %) subjects in Group A subjects compared with 3.3 % (95 % CI: 0.9–8.2 %) in Group B. During the 43-day follow-up period after the second dose, the observed incidence of any type of rash was 5.8 % (95 % CI: 2.4–11.6 %) in Group A and 2.5 % (95 % CI: 0.5–7.1 %) in Group B (Fig. 2).

At least one unsolicited symptom was reported in 45.9 % Group A versus 36.9 % Group B subjects during the 43-days post-dose-1 follow-up period. The most common unsolicited symptom in Group A was bronchitis (12.3 %; *n* = 15) and viral infection with fever in Group B (9.0 %; *n* = 11). Over the 43-day post-dose 2 follow-up period, unsolicited symptoms were recorded in 28.9 and 34.7 % of subjects in Groups A and B, respectively. The most common symptom was bronchitis (6.6 % [*n* = 8] and 9.1 % [*n* = 13], respectively).

Seven subjects, two in Group A and five in Group B recorded the following SAEs: otitis media and laryngitis (each accounting for two cases), gastroenteritis, concussion, bronchitis, breath holding attack, mastoiditis and sinusitis. One child experienced three SAEs (mastoiditis, otitis media and sinusitis). None of the SAEs were fatal and all were considered unrelated to vaccination.

## Discussion

The European Medicines Agency has advised vaccine manufacturers to gradually eliminate the use of blood derived products of human origin because of a theoretical risk of contamination [27, 32]. This phase II study conducted in the Czech Republic and Hungary compared the immunogenicity and safety of a varicella vaccine without HSA (developed to comply with the EMA guidance) with that of the licensed varicella vaccine in children below two years of age. After one dose, the immune responses elicited by the varicella vaccine without HSA were non-inferior to those elicited by the licensed varicella vaccine, i.e. the LL of the two-sided standardized asymptotic 95 % CI for the between-vaccine IFA-measured GMT ratio was ≥0.5 and for the ELISA-measured GMC ratio was ≥0.67.

One of the most important characteristics for paediatric vaccine development is safety, especially as it is essential to maintain high vaccination coverage to control disease.

We utilized two assays in this study: IFA, the serologic method used to license the original varicella vaccine and ELISA. The latter test has improved performance characteristics over IFA, i.e. ELISA is more sensitive and can detect lower titres in samples [33, 34]; data from this study could also be used to support a switch to ELISA [33, 34]. In the current study, 43 days after both doses of each vaccine were administered, the seroconversion/seroresponse rates for anti-VZV antibodies were above 98 %, irrespective of which assay was used. These rates are consistent with pre-licensure European studies with the licensed varicella vaccine in which age-stratified children (15 months–2 years and 2–6 years) achieved average seroconversion rates of 97 % [35] and a more recent study from Indonesia where children aged 10 months–12 years demonstrated a seroconversion rate of 97 % to the licensed varicella vaccine [36].

A numerical (but not statistically significant) increase in the incidence of fever was observed following the administration of the first dose of the vaccine without HSA as compared to the HSA-containing vaccine. This difference appeared to be driven by an increase in fever in the group receiving the vaccine without HSA as compared to the HSA-containing group within eight days post-vaccination. The difference was only observed in low grade fever; no difference in high grade fever (>39 °C) was noted. It is unlikely that this increase in low grade fever might have any significant clinical implications.

The results of this study should be interpreted with caution. First, the comparison between IFA and ELISA assays was not an objective of this study and therefore is primarily descriptive. Second, the sample size was not calculated for the analysis of safety and while we discuss the differences in fever rates between the two vaccines, we cannot draw strong conclusions based on it. Third, administering varicella vaccines with other routine vaccines would have presented a more complete view and further research may be required.

## Conclusion

The first dose of a new varicella vaccine without HSA was immunologically non-inferior to the licensed varicella vaccine. Two doses of the varicella vaccine had an acceptable safety profile in children aged 11–21 months in the Czech Republic and Hungary. We utilized two assays in this study because the ELISA test has reputed improved performance characteristics over the IFA and to support a future switch to ELISA.

## Abbreviations

ATP: According-to-protocol
CI: Confidence interval
ELISA: Enzyme linked immunosorbent assay
GMC: Geometric mean concentration
GMT: Geometric mean titre
HSA: Human serum albumin
HZ: Herpes zoster
IFA: Immunoflourescence assay
LL: Lower limit
SAE: Serious adverse events
TVC: Total vaccinated cohort
VZV: Varicella-zoster virus
